# Facile Preparation and Characterization of Carbon Fibers with Core-Shell Structure from Graphene-Dispersed Isotropic Pitch Compounds

**DOI:** 10.3390/nano9040521

**Published:** 2019-04-03

**Authors:** Dong Hun Lee, Yong-Hwan Choi, Kyong Yop Rhee, Kap Seung Yang, Byung-Joo Kim

**Affiliations:** 1 R&D division, Korea Institute of Carbon Convergence Technology, Jeonju-si, Jeollabuk-do 54853, Korea; leedonghun002@gmail.com (D.H.L.); ebaek241@naver.com (Y.-H.C.); 2 Department of Organic Materials & Fiber Engineering, Chonbuk National University, Jeonju-si, Jeollabuk-do 54896, Korea; 3 Department of Mechanical Engineering, College of Engineering, Kyung Hee University, Yongin-si, Gyeonggi-do 17104, Korea; rheeky@khu.ac.kr; 4 Carbon Composite Laboratory, R&D Center, HPK Inc., Hwaseong-si, Gyeonggi-do 18487, Korea; 5 Department of Polymer & Fiber System Engineering, Chonnam National University, Gwangju 61186, Korea

**Keywords:** pyrolysis fuel oil (PFO), isotropic pitch, graphene, carbon fiber

## Abstract

In this study, isotropic pitch-based carbon fibers were prepared from a mixture of petroleum residue and graphene nanoplatelets with different contents. The softening point and synthetic yield of synthesized isotropic pitches were analyzed and compared to characterize the nature of the pitches. The surface and thermal characteristics of the fibers were observed using scanning electron microscopy and thermogravimetric analysis (TGA), respectively. From the results, it was observed that the prepared carbon fibers had an interesting core-shell structure. In the TGA analysis with air, the carbon fiber having 0.1 wt.% of graphene showed a higher residue yield than that of the sample having 1.0 wt.% of graphene. This result can be explained due to the graphene being placed on the surface region of the carbon fibers and directly helping to increase the surface area of the carbon fibers, resulting in rapid oxidation due to the enhanced contact area with oxygen.

## 1. Introduction

Carbon fiber (CF) can be prepared from various precursors, such as gases (benzene [1], ethane [2], and methane [3]), polymers (cellulose (rayon), polyacrylonitrile (PAN), polyvinylchloride, and phenol resin [4]), and pitches (isotropic and mesophase pitch). Carbon fiber is considered a useful reinforcement for composites due to its excellent properties, such as its high modulus, dimensional stability, and excellent thermal and electrical conductivities [5,6,7,8,9]. Commercial production has been achieved from only three kinds of precursors: PAN, rayon, and pitches [10]. The pitch-based CFs can be further classified into two types: isotropic pitch and anisotropic (or mesophase) pitch [11,12]. Isotropic pitch-based CFs are widely used for general performance applications because isotropic pitch is easy to spin, and its physicochemical properties can be easily controlled during the carbonization process [13]. Graphene has become one of the most important nanomaterials, and many researchers have been continuing various studies using it in various fields [14,15,16]. Recently, a variety of studies to combine graphene and other materials are underway [17,18,19,20] in attempts to unlock synergetic effects of two materials. From the point of view of mechanical applications, Ji et al. [17] have reviewed graphene/polymer composite fibers. These composite fibers usually exhibit enhanced mechanical, thermal, conductive, and antibacterial properties. In the aspect of energy storage applications, He et al. [18] have made microporous carbon/graphene composites using coal tar pitch and graphene oxide via KOH activation. These composites exhibit a high specific capacitance, good rate performance, and excellent cycle stability due to the good electrical properties that result from the addition of the proper content of graphene. Cheng et al. and Ma et al. [19,20] have studied the co-carbonization behavior of petroleum pitch/graphene oxides. Such composites form different anisotropic structures depending on the content of graphene. However, the present works focus on the properties of graphene/polymer composite fibers or electrical properties of graphene/pitch based porous materials; there has been no report on surface morphology change of graphene-dispersed isotropic pitch-based fiber. In this study, graphene-dispersed isotropic pitch was synthesized, and CF was prepared using it. Various contents of graphene were added to the pitch during the synthesis step to observe the effects of graphene content on the morphology of the CFs. The morphology change and surface properties of the CFs with addition of graphene were observed using scanning electron microscopy (SEM), transmission electron microscopy (TEM), atomic force microscopy (AFM), X-ray photoelectron spectroscopy (XPS), Raman spectroscopy, and X-ray diffraction analysis (XRD); the thermal and electrical properties were also characterized.

## 2. Materials and Methods

### 2.1. Materials

The isotropic pitch was synthesized from pyrolysis fuel oil (PFO)-based materials (PFO^#^), which were thermally fractionated at 360 °C. The phase of PFO^#^ is solid and has heavy molecular weight fractions of PFO via thermal separation. XGnP® Graphene Nanoplatelets Grade C650 (XG Sciences Inc., Lansing, MI, USA) was selected for the graphene source. N-methyl-2-pyrrolidone anhydrous grade (NMP, Sigma-Aldrich Co., St. Louis, USA) was used as a dispersion agent for the graphene.

### 2.2. Synthesis of Isotropic Pitch Using PFO^#^

Heat treatment was applied for the synthesis of isotropic pitches without any catalyst. A 500 mL Pyrex flask containing 30 g of PFO^#^ was placed in the reactor; it was heated to 280 or 300 °C at a 5 °C/min heating rate, held at that temperature for a certain time, and finally cooled to room temperature. While the heat treatment was underway, the reactant was bubbled with nitrogen at 800 mL/min and stirred at 400 rpm at the same time.

### 2.3. Synthesis of Isotropic Pitches Using PFO^#^ and Graphene

Graphene was put into beakers with various PFO^#^ weight%’s with 30 g of NMP and sonicated for 120 min to disperse the material. The 500 mL flask containing 30 g of PFO^#^ was placed in the reactor and the dispersed graphene solution was poured into the flask. A two-step heat treatment was used for the synthesis. First, the mixture was heated to 300 °C at 5 °C/min, held for 360 min, and cooled down. Then, it was heated again to 350 °C at 5 °C/min and held at that temperature for 240 min and before cooling to room temperature.

### 2.4. Carbon Fiber Preparation

**Figure 1:**
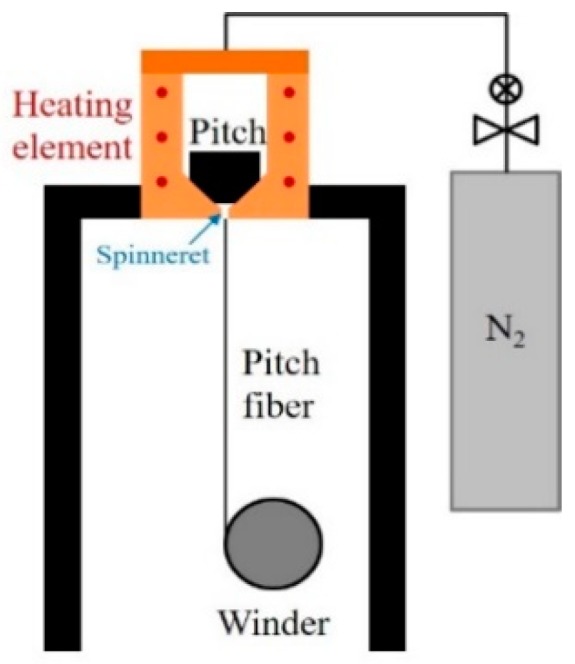
Melt-spinning instrument scheme.

Synthesized pitches (with or without graphene) were deposited in a stainless-steel chamber equipped with a spinneret (0.5 × 0.5 mm, L/D = 1) and spun into fiber at 300 m/min speed while applying pressurized nitrogen gas (Figure 1). The spinning temperature was controlled in a range 40 to 50 °C higher than the temperature of the softening point of the pitches [21]. The spun fibers were stabilized in an oven under air atmosphere, followed with a two-step program of heating at 1 °C/min from room temperature to 300 °C and holding for 60 min at the same temperature. The stabilized fibers were carbonized in a horizontal furnace heated under a nitrogen atmosphere at 5 °C/min up to 1000 °C, at which temperature, they were held for 60 min.

### 2.5. Characterization of Materials and Products

The yield of synthesized pitches was calculated using Equation (1), and the softening points of synthesized pitches were measured using a Mettler FP 90(Mettler Toredo, Columbus, Ohio, USA) by following the directions of the ASTM (American Society for Testing and Materials) D3104 standard test method.

(1) $$\mathrm{Yield}\left(\%\right)=\frac{Weight\;of\;the\;product}{Weight\;of\;the\;reactant}\times 100$$

The thermal properties of the samples were analyzed using an SDTA 841e – TGA analyzer (Mettler Co.) in the temperature range of 30 °C to 800 °C at a heating rate of 10 °C/min in an air atmosphere. 

The surface structure and functional groups of the fibers were analyzed using SEM (S-4700, Hitachi, Tokyo, Japan), AFM (XE-70, Park systems. Co., Suwon, Korea), XPS (PHI 5000 Versa Probes II, ULVAC-PHI, Inc., Kanagawa, Japan), and TEM (JEM-2010, JEOL, Co., Tokyo, Japan). In order to prepare the TEM sample, the carbon fibers were molded into an epoxy resin. The samples were cut using a focused ion beam (FIB, JIB-4601F, JEOL, Co., Tokyo, Japan). The microstructural property was analyzed using Raman spectroscopy (LabRAM HR800, Horiba, Co., Kyoto, Japan) and XRD (X’pert pro Powder, Malvern PANalytical., Eindhoven, Netherlands). The electrical resistivity of the carbon fibers was measured using a Loresta GP resistivity meter (MCP-T610, Mitsubishi Chemical Co., Kanagawa, Japan) connected with a four-point-probe (MCP-TP03P, Mitsubishi Chemical Co., Kanagawa, Japan). A tubular furnace with an alumina tube was used for the oxidation of the carbon fibers to observe different weight loss behaviors via surface oxidation due to the different morphologies of the fibers. Material was heated to 500 °C at 20 °C/min and held at the same temperature for 40 min with 100 mL/min of nitrogen flow. Then, the atmospheric gas was shifted from nitrogen to air. Finally, system was held for 20 min in the same conditions for both samples.

## 3. Results and Discussion

The sample preparation ID of PFO^#^280(240)-800 indicates PFO^#^ was manufactured using the following heat treatment conditions: ‘280′ and ‘(240)’ are the holding temperature and time; ‘800′ is the flow rate of nitrogen atmosphere. The data about synthesis yield and softening points are listed in Table 1. The PFO^#^ was observed as a solid petroleum residue with a softening point in the range of 162.0 to 172.7 °C. As the reaction temperature and time increased, the yield and the softening point changed. When the reaction temperature increased, the yield showed a decrease, and the softening point exhibited an increase. In the case of the increase in holding time, two factors (softening point and synthesis yield) showed the same trend when the reaction temperature increased. This is probably due to the decrease in the low molecular weight fractions using a heat treatment and the increase in average molecular weight. When graphene was added, it was possible through the heat treatment to produce a pitch having a softening point similar to that in the case of the material not containing graphene. 

**Figure 2:**
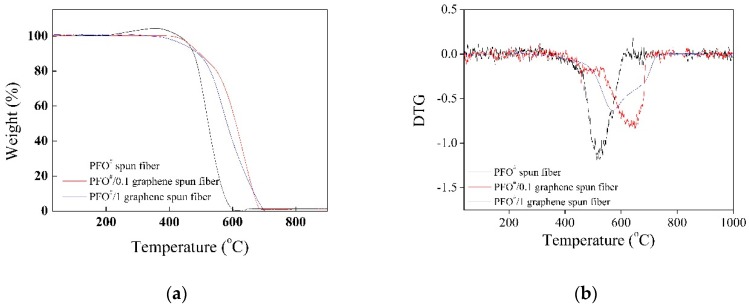
Thermal analysis of PFO^#^-based fibers before and after graphene addition; (**a**) TGA, (**b**) DTG.

Figure 2 shows the TGA and derivative of the TGA (DTG) data of PFO^#^-based fibers (nitrogen atmosphere, 50 mL/min of feeding rate). PFO^#^/graphene-based fibers exhibited a lower weight loss than that of the PFO^#^-based fibers. The weight of the PFO^#^-based fibers decreased rapidly around 500 °C. However, the PFO^#^/graphene-based fibers showed better thermal resistance (they showed a similar drop point around 550–600 °C). It seems that the addition of graphene significantly increased the thermal stability of the pitch fiber.

Generally, pitch-based spun fibers are highly brittle and difficult to handle. Thus, in this work, they were stabilized in an oven under an ambient condition by heating them from room temperature to 300°C at a heating rate of 1 °C/min and then maintaining this temperature for 60 min [22]. Finally, the stabilized fibers were carbonized by heating up to 1000 °C at a heating rate of 5 °C/min in an inert atmosphere. The mass change of the pitch-based fibers is listed in Table 2. It is found that a significant weight was gained in the stabilized fibers (≈10.5%) because oxygen atoms diffuse into the fibers to induce thermosetting [23]. This crosslinking reaction helps the fibers maintain their shape and allows them to be carbonized in the following process without inter-filament fusing [24]. It is interesting to note that the fibers having graphene exhibit lower stabilization yield than that of the neat pitch-based fiber. This indicates that graphene is not oxidized or is less oxidized at the stabilization condition. Moreover, it can be recognized that the graphene played the role of a gas barrier, preventing oxygen diffusion in the fiber due to the severe difference of the stabilization yield.

**Figure 3:**
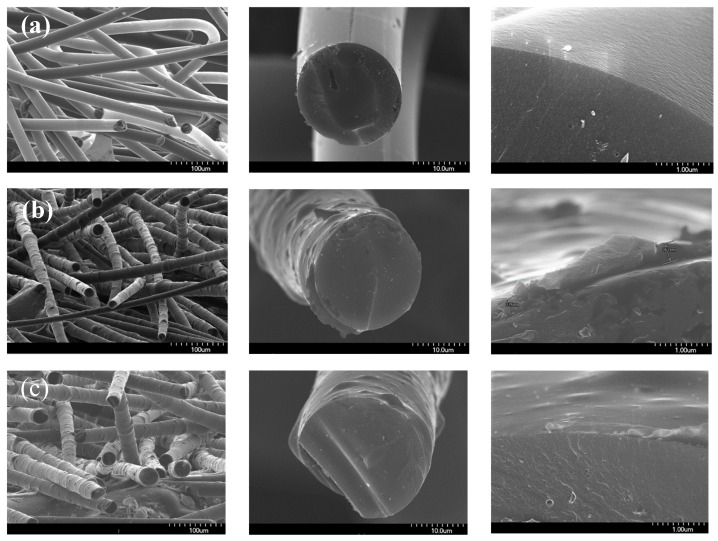
SEM images of PFO^#^-based carbon fibers; (**a**) PFO^#^, (**b**) PFO^#^/0.1 graphene, and (**c**) PFO^#^/1.0 graphene.

Figure 3 presents SEM images of the PFO^#^-based carbon fibers. The neat pitch-based carbon fiber (Figure 3a) had smooth morphology and exhibits a range of diameters of 20.7–23.7 µm. There were no distinctive defects on the surfaces of the fibers. However, some voids were observed in the SEM micrographs of the surface and cross-sectional area of the PFO^#^/graphene-based CFs, and the skin area of the fibers showed a unique core-shell structure. It is considered that the graphene added during the synthesis of pitch did not directly participate in the reaction, but was placed at the outside of the fiber during the spinning process as a result of the different rheological properties of the pure pitch and the pitch/graphene-mixed domain (during spinning, the core areas of the fibers were exposed to high flow compared to the outer side; this can cause extraction of graphene added to the outer side, resulting in the formation of a core-shell structure).

**Figure 4:**
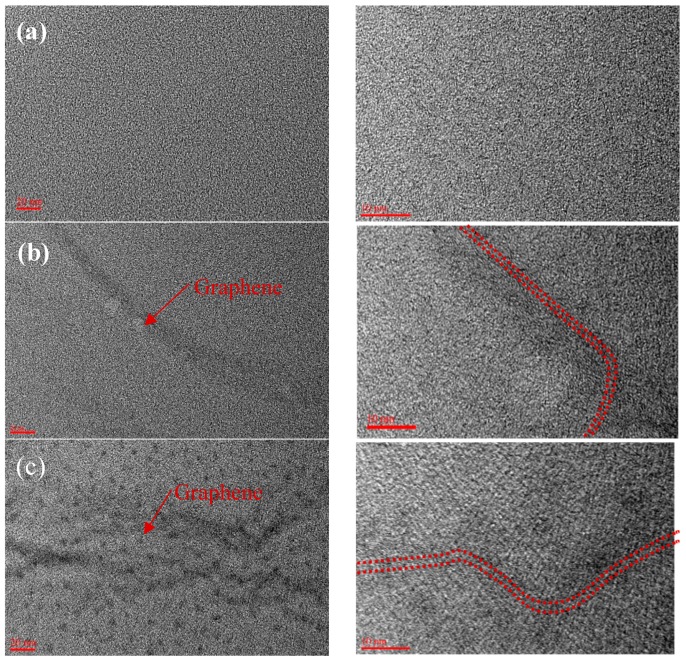
TEM images of PFO^#^-based carbon fibers: (**a**) PFO^#^ and (**b**,**c**) PFO^#^/0.1 graphene.

Longitudinal TEM images of PFO^#^-based carbon fibers are shown in Figure 4. Concerning the longitudinal TEM images, the structure of the PFO^#^-based carbon fiber displayed a random microtexture of carbon microcrystallite. The structure of the PFO^#^/0.1graphene-based carbon fiber displayed a random microtexture of carbon microcrystallite with crumpled graphene sheets. In the high magnification images, the graphene sheet displayed a well-aligned carbon crystallite microtexture.

**Figure 5:**
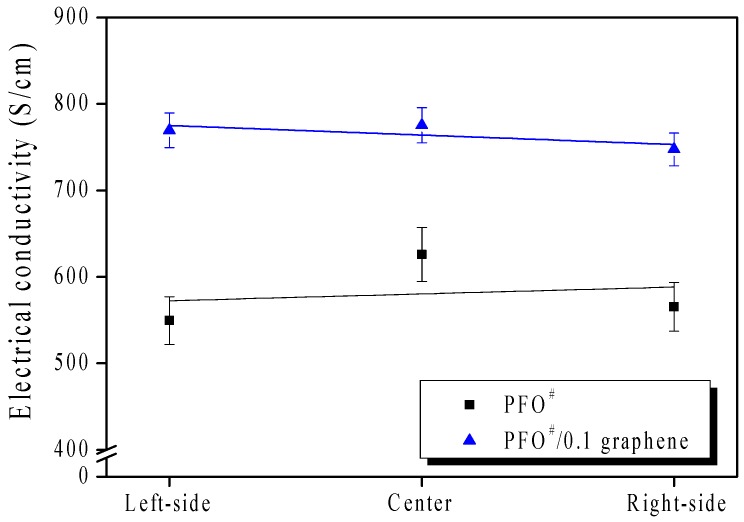
Electrical conductivity of PFO^#^-based carbon fibers.

Figure 5 confirms that the electrical conductivity of PFO^#^-based carbon fibers showed a slight increase with the addition of the graphene. The electrical conductivity of the carbon fiber was considered to be affected by the graphene, which oriented in the longitudinal direction as shown in Figure 4. 

**Figure 6:**
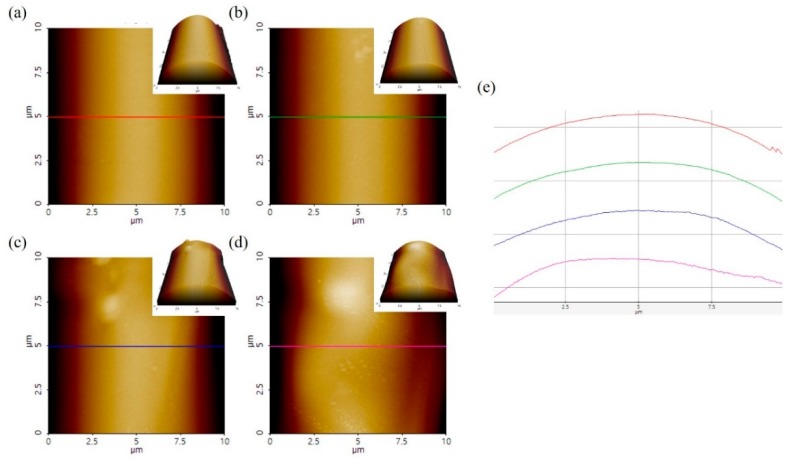
AFM surface morphology of PFO#-based carbon fibers; (**a**,**b**) PFO^#^, (**c**,**d**) PFO^#^/0.1graphene, and (**e**) height pattern along the color lines.

AFM topographic images of PFO^#^-based carbon fibers and height profiles derived from AFM are presented in Figure 6. An absolute comparison using AFM was impossible due to the difference in diameter of the fibers. However, the AFM images show particles on the surface after addition of graphene, and the height profiles were also significantly changed.

The diffraction patterns of the PFO^#^-based carbon fibers show two broad (002) diffraction peaks at around 2θ = 23°, with another (10*l*) diffraction peak at around 2θ = 44°, indicating a graphitic structure [25,26]. The inter-planar spacing d_002_ was calculated using the Bragg equation from the intensity of the diffraction peak. Lateral size (*L_a_*) and the stacking height (*L_c_*) of the crystallite were determined using Equation (2,3) [25,26]:

(2) $$L_{a}=1.84\lambda /A_{a}\cos\beta_{a}$$

(3) $$L_{c}=0.89\lambda /A_{c}\cos\beta_{c}$$

where *λ* is the wavelength of X-rays used, *A_α_* and *A_c_* are the full half width of the (10*l*) and (002) peaks, and *β_α_* and *β_c_* are the corresponding scattering angles.

**Figure 7:**
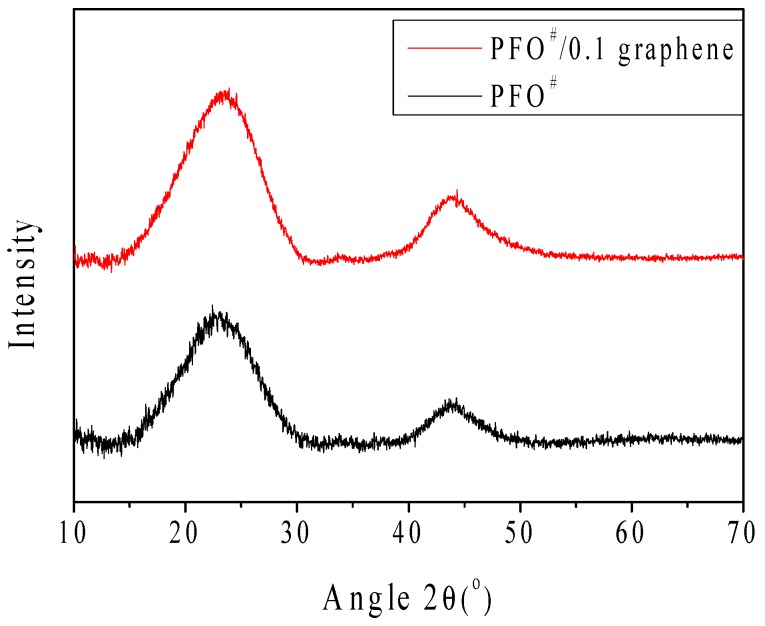
XRD patterns of PFO^#^-based carbon fibers; PFO^#^, PFO^#^/0.1 graphene.

Figure 7 and Table 3 present the XRD results of the PFO^#^-based carbon fibers. It can be seen that the XRD peak intensities of carbon fibers with 0.1 wt.% of graphene were higher than those without graphene, and the addition of graphene reduces the lateral size (*L_α_*) but increased the stacking height (*L_c_*). It was also interesting to note that the centers of the (002) and (101) peaks were shifted to the right side, indicating that graphitization was accelerated by the addition of graphene to the pitch.

**Figure 8:**
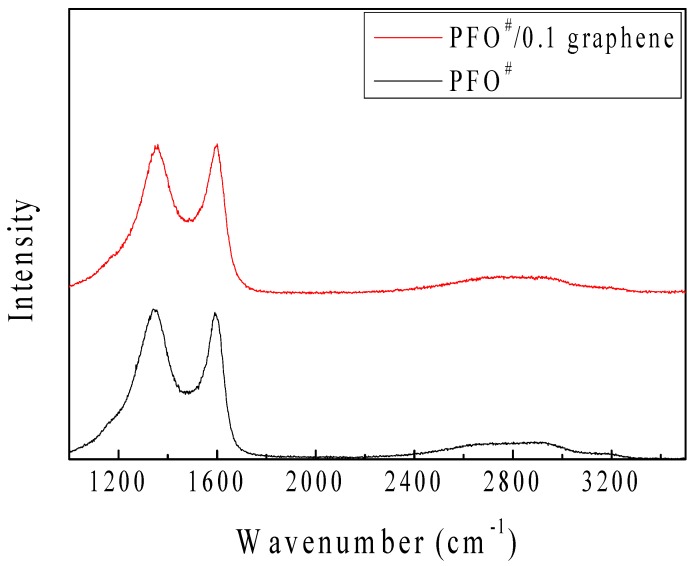
Raman spectra of PFO^#^-based carbon fibers; PFO^#^, PFO^#^/0.1 graphene.

Figure 8 and Table 4 present the Raman spectroscopy results of the PFO^#^-based carbon fibers. Strong peaks can be seen in the range of 1300–1600 cm^−1^. These Raman spectra exhibited two large peaks: one near 1330 cm^−1^, which was a D peak from amorphous structures of carbon, and another near 1580 cm^−1^, which was a G peak from the graphitic structures of carbon [27,28]. The D-band peak position of carbon fibers shifted from 1346 to 1359 cm^−1^, and the R-value (defined as I_D_/I_G_) decreased from 2.39 to 2.28. These results can be explained as showing that there was a shift toward a higher wavelength number, which meant greater ordering of the PFO^#^/0.1 graphene-based carbon fiber [29].

**Figure 9:**
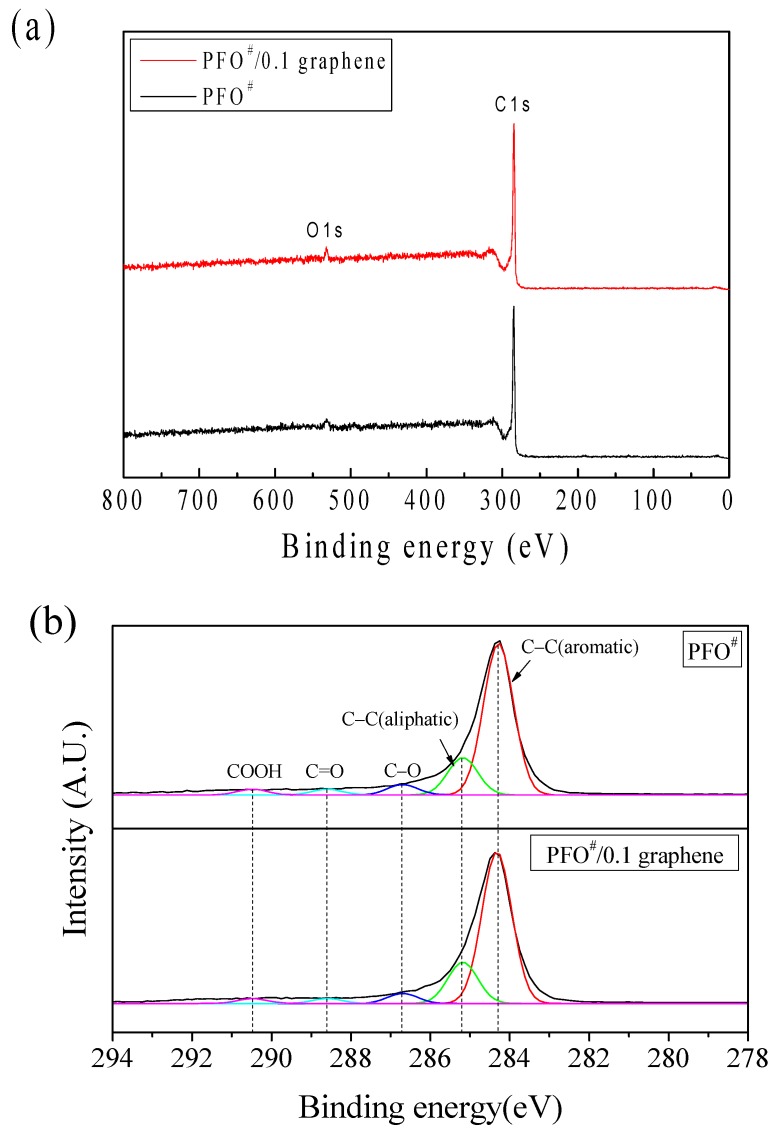
(**a**) Wide-scan XPS spectra and (**b**) C_1s_ XPS spectra of PFO^#^-based carbon fibers.

In the wide-scan XPS spectra (Figure 9a), the surface of PFO^#^-based carbon fibers can be seen to contain the expected two elements (C and a small amount of O), because carbon fibers were formed via carbonization at 800 °C in the nitrogen condition. The C_1s_ spectra (Figure 9b) were used to analyze the types and amounts of functional groups of carbon. These results were fitted into five peaks at 284.3, 285.2, 286.7, 288.6, and 290.5 eV, which correspond to C–C (aromatic), C–C or C–H (aliphatic), C–O, –C=O, and –COOH [30,31,32,33], respectively. The area ratios of aromatic carbon bonds/other carbon bonds, which depends on the degree of disorder of the carbon materials [31], were calculated from Figure 9b and are shown in Table 5. It is clear that the ratio for PFO^#^/0.1 graphene-based carbon fibers was larger than that of PFO^#^-based carbon fibers. This result seems to derive from the more ordered structure shown in the Raman analysis.

**Figure 10:**
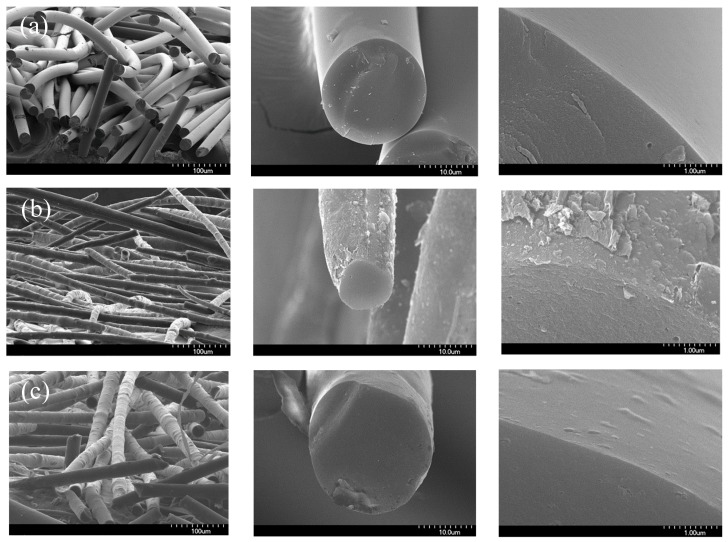
Oxidized PFO^#^-based carbon fibers; (**a**) PFO^#^, (**b**) PFO^#^/0.1 graphene, and (**c**) PFO^#^/1.0 graphene.

Table 6 and Figure 10 present the oxidation results of each carbon fiber. After oxidation, the surface of the carbon fibers was distinctively changed. The surface of PFO^#^-based carbon fibers (Figure 10a) was smoother than that shown in Figure 3a, but in the PFO^#^/graphene-based carbon fibers (Figure 10b,c) the destruction of the core-shell structure during the oxidation process can be observed. It appears that graphene, which was a shell, competitively reacted with oxygen atoms, and this was related to a decrease in oxidation yield when the graphene content increased (Table 6).

These oxidation results are direct proof that the core-shells were formed by graphene addition to the pitch precursors. Moreover, it was possible to obtain functional fibers with high specific surface area using this core-shell structure.

The carbon fibers fabricated in this work were identified as having a higher surface area and better electric conductivity than virgin carbon fibers. Usually, a high surface area of fillers can cause good mechanical adhesion with a polymer matrix in a composite system. Recently, research to apply carbon fibers has been used to fabricate polymer-matrix 3D printing wires [34,35]. However, low adhesion strength between carbon fibers (or other carbonaceous materials) and thermoplastic matrix has been a critical obstacle to commercialization. The high surface area of carbon fibers can be an excellent solution to enhance the mechanical interfacial strength between fibers and thermoplastic matrices.

Moreover, with the development of the shale gas industry, the importance of proton-exchange-membrane-fuel-cells (PEMFC) has been steadily increasing. The gas-dispersion-layer (GDL) in a PEMFC is normally made of carbon fibers due to their excellent mechanical properties, chemical resistance, and electric conductivity. Carbon fibers with a core-shell structure, like those prepared in this work, can be candidate materials for GDL [36,37,38]. Additionally, this kind of carbon fiber can be easily activated by further oxidation in steam and to have porous structures that will maintain higher electric conductivity compared to that of conventionally activated carbon fibers. This means that carbon fibers with core-shell structures can be used as electrode materials for energy storage devices such as fuel cells and supercapacitors.

## 4. Conclusions

In this study, using various thermal treatments, isotropic pitches/graphene precursors were prepared from compounds of petroleum residue and graphene-dispersed solution. As the reaction temperature and time increased, the yield decreased, and the softening point increased. The addition of graphene increased the thermal stability, surface roughness, and ordered structures of the pitch fibers. Isotropic pitch-based carbon fibers, which were prepared from petroleum residue and graphene-dispersed solution, showed core-shell structures. When 0.1 wt.% and 1.0 wt.% of graphene were added, the oxidation yields were 86.6% and 52.1%, respectively, which seem to be due to the acceleration of the oxidation reaction due to the presence of graphene at high temperature. Carbon fibers with a core-shell structure, which have a high surface area, could be used in C/C composite materials, and would result in good adhesion with the polymer matrix. Carbon fibers with more ordering of structures via the addition of graphene lead to improved electrical conductivity, such that they could be used in electrode materials to obtain enhanced electrical conductivity.

## Figures and Tables

**Table 1:** Yields and Softening Points of Pitches Synthesized as Functions of Thermal Treatment Conditions and Graphene Addition.

Sample	Yield (%)	Softening Point (°C)
PFO^#^	-	167.4 ± 5.4
PFO^#^ 280(240)-800	83.3	190.3 ± 0.2
PFO^#^ 300(120)-800	48.3	274.4 ± 6.0
PFO^#^ 300(240)-800	45.0	319.5 ± 8.1
PFO^#^/0.1 graphene 300(360)350(240)-800	50.6	285.6 ± 4.9
PFO^#^/1.0 graphene 300(360)350(240)-800	54.5	277.6 ± 4.8

Note: Each sample was measured two times.

**Table 2:** Process Yields of PFO^#^-based Fibers as Functions of Thermal Treatment Conditions and Graphene addition.

Sample	Stabilization Yield (%)
PFO^#^ 300(240)-800	110.5
PFO^#^/0.1 graphene 300(360)350(240)-800	109.6
PFO^#^/1.0 graphene 300(360)350(240)-800	107.7

**Table 3:** Crystallite size results of PFO^#^-based carbon fibers: PFO^#^, PFO^#^/0.1 graphene.

Sample	002 Peak				10*l* Peak		
	2θ	FWHM	d_002_ (Å)	*L_c_* (Å)	2θ	FWHM	*L_a_* (Å)
PFO^#^	22.95 ± 0.02	7.54	3.73	13.25	44.05 ± 0.06	4.97	35.25
PFO^#^/0.1 Graphene	23.08 ± 0.01	7.73	3.83	17.95	44.24 ± 0.03	6.32	27.74

**Table 4:** Raman Spectra Results of PFO# and PFO#/0.1 Graphene-Based Carbon Fiber.

Sample	Peak Position		Peak Intensity		R-Value (I_D_/I_G_)
	D Band	G Band	D Band	G Band	
PFO^#^	1346.28 ± 0.76	1588.45 ± 0.50	574.28	570.73	2.39
PFO^#^/0.1 Graphene	1359.43 ± 0.84	1591.63 ± 0.52	384.16	396.21	2.28

**Table 5:** Area Ratios of Chemical Bonding Peaks from C_1s_ XPS Spectra.

	Peak Area		Area Ratio
	Aromatic Carbon	Other Carbon	
PFO^#^	5135.04	3225.53	1.59
PFO^#^/0.1 graphene	4596.05	2515.76	1.83

**Table 6:** Yields of Carbon Fibers after Oxidation at 500 °C in Air.

Sample	Yield (%)
PFO^#^ 300(240)-800	89.1
PFO^#^/0.1 graphene 300(360)350(240)-800	86.6
PFO^#^/1.0 graphene 300(360)350(240)-800	52.1

## References

[1] Jayasankar M., Agarwal N., Chand R., Gupta S.K., Kunzru D. (1996). Vapor grown carbon fibers from benzene pyrolysis: Filament length distributions. Carbon.

[2] Thornton J.M., Walker S.G. (2009). Catalytic carbon deposition on three-dimensional carbon fiber preforms using alkane gas feedstocks. New Carbon Mater..

[3] Sacco A., Thacker P., Chang T.N. (1984). The initiation and growth of filamentous carbon from alpha-iron in H_2_, CH_4_, H_2_O, CO_2_, and Co Gas-Mixtures. J. Catal..

[4] Chen Y.J. (2017). Activated Carbon Fiber and Textiles.

[5] Northolt M.G., Veldhuizen L.H., Jansen H. (1991). Tensile deformation of carbon fibers and the relationship with the modulus for shear between the basal planes. Carbon.

[6] Kumar S., Anderson D.P., Crasto A.S. (1993). Carbon fibre compressive strength and its dependence on structure and morphology. J. Mater. Sci..

[7] Edie D.D., Fain C.C., Robinson K.E., Harper A.M., Rogers D.K. (1993). Ribbon-shape carbon fibers for thermal management. Carbon.

[8] Hong S.H., Korai Y., Mochida I. (1999). Development of mesoscopic textures in transverse cross-section of mesophase pitch-based carbon fibers. Carbon.

[9] Hong S.H., Korai Y., Mochida I. (2000). Mesoscopic texture at the skin area of mesophase pitch-based carbon fiber. Carbon.

[10] Peebles L.H. (1995). Carbon Fibers: Formation, Structure, and Properties.

[11] Wazir A.H., Kakakhel L. (2009). Preparation and characterization of pitch-based carbon fibers. New Carbon Mater..

[12] Mora E., Blanco C., Prada V., Santamaria R., Granda M., Menendez R. (2002). A study of pitch-based precursors for general purpose carbon fibres. Carbon.

[13] Lee H.M., Kwac L.K., An K.H., Park S.J., Kim B.J. (2016). Electrochemical behavior of pitch-based activated carbon fibers for electrochemical capacitors. Energy Convers. Manag..

[14] Geim A.K. (2009). Graphene: Status and prospects (Review). Science.

[15] Rao C.N.R., Sood A.K., Subrahmanyam K.S. (2009). Govindaraj, Graphene: The New Two-Dimensional Nanomaterial. Angew. Chem. Int. Ed..

[16] Xu Z., Gao C. (2015). Graphene fiber: A new trend in carbon fibers. Mater. Today.

[17] Ji X., Xu Y., Zhang W., Cui L., Liu J. (2016). Review of functionalization, structure, and properties of graphene/polymer composite fibers. Compos. Part A.

[18] He X., Wang J., Xu G., Yu M., Wu M. (2016). Synthesis of microporous carbon/graphene composites for high-performance supercapacitors. Diam. Relat. Mater..

[19] Cheng Y., Yang L., Fang C., Guo X. (2016). Co-carbonization behavior of petroleum pitch/graphene oxide: Influence on structure and mechanical property of resultant cokes. J. Anal. Appl. Pyrolysis.

[20] Ma Y., Ma C., Sheng J., Zhang H., Wang R., Xie Z., Shi J. (2016). Nitrogen-doped hierarchical porous carbon with high surface area derived from graphene oxide/pitch oxide composite for supercapacitors. J. Colloid Interface Sci..

[21] Yang J., Shi K., Li X., Yoon S.H. (2018). Preparation of isotropic spinnable pitch and carbon fiber from biomass tar through the co-carbonization with ethylene bottom oil. Carbon Lett..

[22] Lee D.H., Choi J.S., Oh y. S., Kim Y.A., Yang K.S., Ryu H.J., Kim Y.J. (2017). Catalytic hydrogenation-assisted preparation of melt spinnable pitches from petroleum residue for making mesophase pitch based carbon fibers. Carbon Lett..

[23] Donnet J.B., Bansal R.C. (1990). Carbon Fibers.

[24] Zhu J., Park S.W., Joh H.I., Kim H.C., Lee S.H. (2013). Preparation and characterization of isotropic pitch-based carbon fiber. Carbon Lett..

[25] Takagi H., Maruyama K., Yoshizawa N., Yamada Y., Sato Y. (2004). XRD analysis of carbon stacking structure in coal during heat treatment. Fuel.

[26] Lu L., Sahajwalla V., Kong C., Harris D. (2001). Quantitative X-ray diffraction analysis and its application to various coals. Carbon.

[27] Endo M., Kim C., Karaki T., Kasai T., Matthews M.J., Brown S.D.M., Dresselhaus M.S., Tamaki T., Nishimura Y. (1998). Structural characterization of milled mesophase pitch-based carbon fibers. Carbon.

[28] Zhu Y., Zhao X., Gao L., Cheng J. (2018). Pyrolysis kinetics and microstructure of thermal conversion products on toluene soluble component from two kinds of modified pitch. Carbon Lett..

[29] Roh J.S., Kim S.H. (2009). Structural Study of the Oxidized High Modulus Carbon Fiber using Laser Raman Spectroscopy. Carbon Lett..

[30] Kim B.H., Lee D.H., Yang K.S., Lee B.C., Kim Y.A., Endo M. (2011). Electron Beam Irradiation-Enhanced Wettability of Carbon Fibers. ACS Appl. Mater. Interfaces.

[31] Boudou J.P., Paredes J.I., Cuesta A., Martìnez-Alonso A., Tascón J.M.D. (2003). Oxygen plasma modification of pitch-based isotropic carbon fibres. Carbon.

[32] Moreno-Castilla C., López-Ramón M.V., Carrasco-Marín F. (2000). Changes in surface chemistry of activated carbons by wet oxidation. Carbon.

[33] Estrade-Szwarckopf H. (2004). XPS photoemission in carbonaceous materials: A “defect” peak beside the graphitic asymmetric peak. Carbon.

[34] Zhong J., Zhou G.-X., He P.-G., Yang Z.-H., Ja D.-C. (2017). 3D printing strong and conductive geo-polymer nanocomposite structures modified by graphene oxide. Carbon.

[35] Zhang H., Yang D., Sheng Y. (2018). Performance-driven 3D printing of continuous curved carbon fibre reinforced polymer composites: A preliminary numerical study. Compos. Part B.

[36] Indayaningsih N., Priadi D., Zulfia A. (2011). Suprapedi, Analysis of coconut carbon fibers for gas diffusion layer material. Key Eng. Mater..

[37] Kannan M.A., Munukutla L. (2007). Carbon nano-chain and carbon nano-fibers based gas diffusion layers for proton exchange membrane fuel cells. J. Power Sources.

[38] Tötzke C., Manke I., Hartnig C., Kuhn R., Riesemeier H., Banhart J. (2011). Investigation of carbon fiber gas diffusion layers by means of synchrotron X-ray tomography. ECS Trans..

